# Norovirus Vaccine: Priorities for Future Research and Development

**DOI:** 10.3389/fimmu.2020.01383

**Published:** 2020-07-07

**Authors:** Susanna Esposito, Nicola Principi

**Affiliations:** ^1^Pediatric Clinic, Department of Medicine and Surgery, Pietro Barilla Children's Hospital, University of Parma, Parma, Italy; ^2^Università degli Studi di Milano, Milan, Italy

**Keywords:** acute gastroenteritis, GI strain, GII strain, norovirus, norovirus vaccine

## Abstract

Soon after its identification, norovirus (NoV) has been indicated as one of the most common causes of outbreaks of acute gastroenteritis (AGE) and sporadic acute diarrhea episodes in subjects of any age. In 2016 the World Health Organization stated that the development of a NoV vaccine should be considered an absolute priority. Unfortunately, the development of an effective NoV vaccine has proven extremely difficult, and only in recent years, some preparations have been tested in humans in advanced clinical trials. In this paper, reasons that justify efforts to develop a NoV vaccine, difficulties encountered during NoV vaccine development, and NoV vaccine candidates will be discussed. In recent years, identification of some NoV antigens that alone or in combination with other viral antigens can induce a potentially protective immune response has led to the development of a large series of preparations that seem capable of coping with the problems related to NoV infection. Epidemiological and immunological studies have shown that multivalent vaccines, including both GI and GII NoV, are the only solution to induce sufficiently broad protection. However, even if the road to formulation of an effective and safe NoV vaccine seems to be definitively traced, many problems still need to be solved before the total burden of NoV infections can be adequately controlled. Whether currently available vaccines are able to protect against all the heterologous NoV strains and the variants of the most common serotypes that frequently emerge and cause outbreaks must be defined. Moreover, as performed clinical trials have mainly enrolled adults, it is mandatory to know whether vaccines are effective in all age groups, including younger children. Finally, we must know the immune response of immunocompromised patients and the duration of protection induced by NoV vaccines. Only when all these problems have been solved will it be possible to establish an effective immunization schedule against NoV infection and calculate whether systematic vaccination can be cost effective.

## Introduction

Soon after its identification in 1972, norovirus (NoV) has been indicated as one of the most common causes of outbreaks of acute gastroenteritis (AGE) and sporadic acute diarrhea episodes in subjects of any age (1). The development of effective rotavirus (RV) vaccines and their introduction in the official pediatric immunization schedules has made, at least in countries where these vaccines have been largely used, the epidemiologic relevance of NoV even more evident (2). Several studies have highlighted the enormous medical, social and economic problems caused by NoV disease and the need for the development of adequate preventive measures against this virus. However, the development of specific antivirals, theoretically useful to interrupt outbreaks or to avoid infection in subjects at increased risk, has been very difficult if not totally impossible for many years due to the lack of adequate cell lines for viral culture and successful animal models for drug evaluation. Although recent advances in culturing human NoV lead us think that, in the future, effective anti-NoV drugs will become available, it is clear that only the availability of safe and effective vaccines can face and definitively solve all the problems related to NoV infection (3). This perspective is strongly highlighted by the decision of the World Health Organization that stated in 2016 that the development of a NoV vaccine should be considered an absolute priority (4). Unfortunately, the development of an effective NoV vaccine has proven extremely difficult, and only in recent years, some preparations have been tested in humans in advanced clinical trials (5). In this paper, reasons that justify efforts to develop a NoV vaccine, difficulties encountered during NoV vaccine development, and NoV vaccine candidates will be discussed.

## Reasons That Justify the Development of a Specific Norovirus (NoV) Vaccine

### High Frequency of Norovirus (NoV) Acute Gastroenteritis

NoV is the most common aetiologic agent of AGE worldwide. Among the 2.7–4 billion diarrhoeal cases that are globally diagnosed every year, ~18% are associated with NoV infection, without significant differences between developed (20%) and developing countries with low diarrhea-related mortality (19%) (6–8). The incidence of NoV diarrhea is, in contrast, slightly lower in high-mortality developing nations (14%), where some bacterial pathogens continue to play a relevant aetiologic role for AGE determination due to the lack of adequate infection control measures. NoV diarrhea is diagnosed in patients of any age. Studies carried out in some developed countries have shown that from 3.8 to 10.4% of the population is affected by NoV disease every year, suggesting that during a lifetime, each individual can suffer from 3 to 8 NoV diarrhoeal episodes (9–14). However, the incidence of disease is higher in younger children and in adults ≥65 years old. This fact highlights that among healthy subjects, those who have physiologically lower defenses are those with the highest risk of NoV AGE and those for whom effective preventive measures are urgently needed. In the UK, incidence rates were found to be significantly higher in children <5 years old than in individuals of any other age group (142.6 cases per 1,000 person-year, 95% confidence interval [CI] 99.8–203.9 vs. 37.6, 95% CI 31.5–44.7, respectively). Moreover, it was shown that in adults ≥ 65 years old, development of NoV disease was two times more common than in those aged 15–64 years (15).

However, it is highly likely that the reported incidences of NoV infection and disease grossly underestimate the true values. In low-income countries, reliable methods used to detect NoV, such as those based on molecular biology, are frequently not available, and most of the diarrhoeal cases, even among those hospitalized, remain aetiologically undefined. The same is true for most of the cases that are too mild to be hospitalized or seen in the emergency room that occur in industrialized countries. On the other hand, even in studies specifically devoted to analyzing NoV epidemiology, asymptomatic cases may not be identified unless systematic periodic stool examination is performed.

### Potential Severity of Norovirus (NoV) Acute Gastroenteritis

Although NoV infection can remain asymptomatic in 20–30% of cases or lead to a mild disease (16), spontaneously resolving in 2–3 days in the majority of patients (17), severe AGE is not rare. NoV is the cause of 10–20% and 1–15% of emergency room visits and hospitalizations in middle/high-income countries and in low-income countries, respectively. Moreover, more than 3% of patients with a NoV diarrhea episode can die, as shown by the evidence that among the 677 million cases diagnosed in 2010 worldwide, 213,515 died (18). As expected, the risk of severe NoV AGE is increased in children <5 years old and in elderly individuals. Moreover, immunocompromised patients are at increased risk. This population is a third group of subjects for whom the availability of a vaccine might be very important, although their condition limits a relevant immune response and protection. Forty-three percent of deaths that occurred in 2010 occurred in children <5 years old (18). Moreover, in elderly people, dehydration leads to intensive care unit admission, and in some cases, death is relatively common (19). Finally, in immunocompromised patients, such as those with solid organ or bone marrow transplantation, NoV infection can become chronic and last from weeks to years. This chronicity leads to prolonged virus shedding and gastrointestinal disease that can become increasingly debilitating and lead to death (20, 21). A study of 123 deaths associated with NoV infection revealed that 10 of these cases occurred in patients immunocompromised because of chemotherapy or transplantation (22).

### Norovirus (NoV) Acute Gastroenteritis and Socioeconomic Burden

The socioeconomic burden of NoV infection is enormous. It has been calculated that in recent years, the global annual mean economic burden of NoV infection was $64.5 billion, of which $4.2 billion were related to the direct health system and $60.3 billion due to societal costs (23). The costs for illness were higher for people ≥55 years old and were mainly related to hospitalization. In contrast, societal costs were higher for children <5 years old, and in this case, parents' productivity losses played the major role in causing economic problems. Interestingly, high-income countries had the highest global health system costs, highlighting that vaccine prevention of NoV infections can play a relevant role worldwide, although the main reasons can vary according to the geographic area and the age of the involved population.

### High Risk of Norovirus (NoV) Infection Transmission

NoV is extremely contagious, and transmission of infection from person to person occurs easily and is difficult to interrupt in the absence of effective prophylactic measures. Vomitus and feces of infected subjects contain a high number of virions, whereas only 10 infectious particles are required to cause AGE. Moreover, NoV has high environmental stability, and shedding after infection usually lasts weeks (24). Transmission occurs generally directly via fecal-oral and vomit-oral pathways from person to person, although it can also be caused by foodborne, waterborne or environmental fomites. Children are the most important source of infection transmission in families and in schools. Outbreaks can occur in healthcare facilities, including nursing homes and hospitals, with dramatic consequences, particularly when younger children, older patients and immunocompromised subjects are involved (25).

## Challenges in the Development of a Norovirus (NoV) Vaccine

### Genetic Diversity of Circulating Norovirus (NoV) Strains

Genetic analysis of the entire genome or individual virus genes has led to the identification of seven NoV genogroups. Moreover, studies of the sequences that encode for the capsid protein VP1 and RNA-dependent RNA polymerase have shown that in each genogroup, several genotypes are included. Presently, over 30 genotypes have been identified. Genogroups I (GI), II (GII), and IV (GIV) are defined as the human NoVs because the NoV AGEs that occur in humans are exclusively due to strains included in these serogroups (26). However, GII is the predominant strain worldwide. An analysis of 16,635 sequences of NoV specimens collected between 2005 and 2016 in Europe, Asia, Oceania and Africa from outbreak investigations and sporadic AGE cases revealed that 91.7% belonged to genotype GII, 8.2% to GI and <0.1% to GIV (27). Genogroups have high genetic diversity. Moreover, genotypes included in a single genogroup can have slight but important differences in antigenic characteristics. Finally, single genotypes may frequently experience point mutations and recombination events that produce antigenically different variants (28).

All the above-mentioned factors explain both the difficulties in finding an effective universal vaccine and the frequent AGE outbreaks due to the same NoV genotype. This last problem has been clearly evidenced with NoV GII.4, the most frequent cause of AGE outbreaks. Since the 1990's, every 2–3 years, a new GII.4 variant has emerged, causing at least 7 NoV AGE epidemics in different geographic areas (29, 30). However, other genotypes can cause epidemics. Among those recently identified, the most common are some viruses derived by a recombination process, such as GII.P16-GII.4 Sidney, GII.P16-GII2, GII.P4 New Orleans-GII.4 Sydney, GII.P12-GII.3 and GI.P6-GI6, which were associated with significant outbreaks in the USA, Asia and Europe (27, 31).

### Poor Knowledge of Characteristics of Immunity to Natural Norovirus (NoV) Infection

Infections due to a NoV included in a genogroup generally do not confer protection to infections due to another genogroup. This phenomenon explains why after attempts to produce vaccines with a single NoV, NoV vaccines are presently based on viruses included in both GI and GII (32). In contrast, adults naturally infected by a NoV genotype develop protective immunity against reinfection with the same genotype. Protection lasts some years but does not seem to be long lasting. It has been reported that after an infection with a given genotype, challenge with the same genotype some weeks later did not lead to infection. However, when the challenge was performed 2–6 years later, symptoms of AGE could occur (33). Similar findings have been reported in children. O'Rayan et al. (34) reported that in the first 18 months of age, children suffer from repeated episodes of NoV infection and that most of them remained asymptomatic. In contrast, symptomatic infection due to GII genotypes was observed only as primary infections or when a previous episode was due to a different GII genotype. However, it is not precisely defined how long the effective protection lasts; if there are differences between adults and children, particularly the youngest; and if antigenic drift plays a role, as in the case of influenza virus, to reduce protection evoked by previous infections with the same genotype. On the other hand, challenge studies are carried out with viral inocula significantly higher than those needed to cause natural infection, and this difference can modify the immune response, leading to incorrect conclusions.

### Lack of Immune Correlates of Protection

The availability of an immune marker able to indicate whether an individual is protected from an infection and related disease can significantly contribute to the development of a vaccine by benefiting evaluation of potential vaccine efficacy. Unfortunately, no reliableimmune correlate of protection from NoV infection and disease valid for all the people has been identified. The use of total serum NoV-specific antibody levels was debated. After infection, NoV-specific antibody concentrations tend to increase in most patients (33), and in an epidemiological study, serum NoV-specific antibody levels were inversely correlated with protection from NoV AGE development (35). However, in general, pre-infection antibody levels were not associated with protection (36).

It is highly likely that some methodological limitations of the above-cited studies led to these conflicting conclusions. Most of the data have been collected before it was known that genetics plays a fundamental role in conditioning susceptibility to NoV infection. If subjects that were genetically resistant to NoV and with no immune response to NoV exposure had been enrolled, the final results would inevitably have been negatively conditioned. Recent studies have shown that ~20–30% of the general population is resistant to NoV infection. Resistance is genotype and even strain dependent and is attributed to the human histo-blood group antigens (HBGAs), mainly those encoded by the FUT2 (Secretor), the FUT 3 (Lewis) and the ABO genes. As these antigens are attachment cofactors for NoV and are essential for NoV entry into surface epithelial cells, missense and non-sense mutations of FU2, FUT3, or ABO genes are associated with the inability of NoV to exert its virulence by causing infection and AGE. FUT2 is the best-studied gene, and several polymorphisms with known effects on secretor status have been identified, many of which are population specific. Generally, people with an absence or a poor presence of the FUT2 enzyme are partially or totally resistant to GI.1 and GII.4 NoVs, although they can remain susceptible to infections due to other NoV genotypes because the mechanism of attachment to host cells can vary among the various NoV genotypes (37).

Recently, other potential correlates of protection have been identified. Among them are the serum concentration of HBGA-blocking antibody levels and the total serum NoV-specific IgA antibody levels. This conclusion was reached when susceptibility to NoV infection and disease after exposure to a GII.4 NoV strain was evaluated in a group of adults that had previously received a bivalent NoV vaccine or placebo. It was found that among subjects receiving placebo, the incidence of virus infection and associated illness was lower when prechallenge serum anti-GII.4 HBGA-blocking antibody levels and specific IgA antibody levels were particularly high. Regarding a putative correlate of protection for anti-GII.4 HBGA-blocking antibody levels, it was shown that the frequency of moderate-to-severe vomiting or diarrhoeal illness was reduced when the levels of this antibody were >1:500 (38). However, measurement of the HBGA-blocking antibody level to evaluate susceptibility to NoV has been poorly used because no stable FUT2-overexpressing cell line has been available. Recently, a FUT2-overexpressing cell line-based surrogate neutralization assay for NoV vaccine evaluation was constructed (39).

Further markers of protection might be NoV-specific salivary and fecal IgA and NoV-specific memory IgG cells. In a study, it was found that NoV-specific salivary IgA levels before exposure to NoV were inversely correlated with the severity of AGE. NoV-specific fecal IgA levels before challenge were correlated with a reduction in peak viral load and, when measured 7 days after infection, with a reduced duration of virus shedding. Finally, increased numbers of NoV-specific memory IgG cells were associated with protection from AGE (40).

## Norovirus (NoV) Vaccine Development

Three types of NoV vaccines have been developed. Non-replicating virus-like particles (VLPs), P particles, and recombinant adenoviruses have been used (Table 1). All these vaccine development platforms have challenges and limitations. NoV VLPs are structures that mimic the organization and conformation of authentic native viruses but lack the viral genome, potentially yielding safer and cheaper vaccine candidates. In case of NoV, vaccines are derived from the major capsid NoV protein VP1 that spontaneously self-assemble into VLPs when NoV VP1 is expressed on eukaryotic cells. VLPs are antigenically similar to the viral particle and are able to evoke a specific antibody response when administered by both the enteral and parenteral routes, without any risk of infection (41).Although several VLP production platforms may be used to produce VLPs, two are those more frequently used: the baculovirus replicon system and the Venezuelan equine encephalitis replicon system (42, 43). Both are relatively inexpensive and allow robust use of these components for NoV vaccine development. However, baculovirus contaminant may be difficult to remove and host-derived insect cell/baculovirus components may mask the immune response against the desired epitope. P particles are nanoparticles formed by copies of the protruding (P) domain of the NoV capsid protein. They contain all the required elements to interact with the viral receptors. Consequently, they are highly immunogenic and activate both the innate and the adaptive immune system eliciting humoral and cellular immunity. Moreover, they are stable and can be readily produced in *E. coli*. For all these reasons, they are considered potentially effective vaccine candidate. However, the evidence that VLPs can induce a greater T helper type 1 (Th1) and Th2 balanced cross-reactive immune response compared with P-particles makes VLPs preferable for the preparation of NoV vaccines (44). Finally, attempts to use recombinant adenovirus expressing a GI1 or GII4 NoV VP1 have been made (45, 46).

**Table 1:** Main Norovirus vaccines in development.

**Vaccine type**	**Preclinical**	**Phase 1**	**Phase 2**
Virus like particle (VLP)	Vaccine Research Center in Tampere, Finland Combined NoV and Rotavirus (RV) vaccine containing NoV VLPs GII.4 and GI.3 and the oligomeric RV VP6 Studies in mice Institute Pasteur of China A vaccine combining NoV GII.4 and Enterovirus 71 VLPs Studies in animal models	National Vaccine and Serum Institute, China, Intramuscolar bivalent GI.1 and GII.4 Healthy subjects 6 months-59 years	Takeda Intramuscolar Bivalent GI.1 and GII.4 In children > 6 months, adults, people > 60 years, and military recruits
Recombinant adenovirus		Vaxart Oral Monovalent GI.1 or GII.4 or bivalent combined in healthy adults	
P particles	Several Research Centers Vaccines combining NoV VPI P domain with RV, hepatitis E, influenza and astrovirus in order to obtain extensive protection. Studies in animal models		

However, independent of the technique used to develop an immunogenic and protective preparation, the real problem is which genotypes must be included in the vaccine formulation. Initially, considering that the first detected NoV was a GI.1 strain, a vaccine containing this virus was developed. Later, when it was shown that GII.4 was the most common cause of NoV AGE and that the level of cross reactogenicity between GI.1 and GII.4 was low, a combined vaccine was prepared. More recently, other combinations or more complicated vaccines containing additional strains or P particles and viral vectors, including NoV capsid genes, have been developed.

### Norovirus (NoV) Vaccines in Clinical Stages of Development

#### Monovalent Vaccines

An intranasal vaccine containing a GI.1 VLP adjuvanted with monophosphoryl lipid A (MPL) was initially prepared. It was found to induce virus-specific serum antibodies in the majority of vaccine recipients. Moreover, when adults that received two doses of this vaccine were challenged with the virus included in the vaccine and compared with previously unvaccinated subjects, it was shown that the risk of NoV infection and of developing AGE were significantly reduced in vaccine recipients compared to those in controls (61% vs 82%: p=0.05 for infection; 37% vs 69%: p=0.006 for disease) (47). Further studies confirmed the immunogenicity of this vaccine, highlighting its possible use in NoV infection prevention. In particular, it was shown that the immune response to the vaccine was strictly dose dependent and that the frequency of NoV-specific IgG- and IgA-secreting B cells in peripheral blood and the level of antibodies produced by these cells in culture were significantly higher in adults that received 100 μg of GI.1 VLP than in those given 50 μg (48). However, considering the risk that NoV infections due to NoV genogroups other than GI.1 could not be prevented, further research was directed mainly to preparing a combination vaccine.

#### Bivalent Vaccines

Both intranasal and intramuscular bivalent NoV vaccines containing GI.1 and GII.4 VLPs have been developed. A dry powder formulation (GelVac™) with an *in situ* gelling polysaccharide (GelSite™) extracted from *Aloe vera* for nasal delivery was tested in guinea pigs to evaluate safety, immunogenicity and potential antigenic interference. The preparation was found to be safe and well-tolerated and able to induce a dose-dependent immune response, with the production of antibodies capable of inhibiting the binding of the VLPs to pig gastric mucin and without any antigenic interference (49). However, considering that intramuscular administration could induce a more rapid and higher antibody response than theintranasal vaccine, a parenteral vaccine was prepared. It was developed by Takeda Pharmaceutical Company Limited and was based on a Norwalk GI.1 strain VLP that cross-reacts with other GI.1 strains and a consensus of 3 GII strains [2006a (Yerseke), 2006b (Den Haag), and 2002 (Houston)] to increase the protection against GII strains as much as possible (50). This formulation is the NoV vaccine presently in an advanced stage of development.

Preclinical studies have shown that this vaccine was highly immunogenic and that induced antibodies were able to recognize and react against not only homologous genotypes but also heterologous genotypes, such as GI.3, GII.1, GII.3, and GIV.1 (51). Use of this bivalent preparation adjuvanted with MPL and aluminum hydroxide in adults aged 18–83 years in two phase I studies confirmed the immunogenicity of the bivalent vaccine (52, 53). Two doses one month apart were administered. Antibodies against GI.1 and GII.4 increased rapidly, reaching a peak level at ~7 days after the first injection. First-dose seroresponse frequencies ranged from 88 to 100% for the GI.1 antigen and from 69 to 84% for the GII.4 antigen. However, the booster effect of the second dose was modest. In most subjects, a significant increase in HBGA-blocking antibody titres was evidenced, without differences according to age. The clinical efficacy of the bivalent vaccine seemed indicated by the data collected with a double-blind, placebo-controlled trial that enrolled adults aged 18–50 years. These subjects received two doses of placebo or bivalent vaccine 4 weeks apart and were challenged with a GII.4 virus not included in the consensus GII.4 sequence. Monitoring of clinical conditions after challenge revealed that signs and symptoms of NoV disease were less common and less severe in vaccine recipients than in controls, although differences did not reach statistical significance. Severe, moderate or mild vomiting and diarrhea were evidenced in vaccinees at rates of 0, 6, and 20.0%, respectively, compared to rates of 8.3% (*p* = 0.054), 18.8% (*p* = 0.068), and 37.5% (*p* = 0.074), respectively, for controls. Moreover, the modified Vesikari score for AGE severity was reduced in vaccinees from 7.3, calculated in controls, to 4.5 (*p* = 0.002). Finally, a lower number of subjects receiving the vaccine had virus shedding by day 10 after challenge than that of controls (22.4% of vaccinees vs. 36.2% of controls; *p* = 0.179). No severe adverse event was observed (54).

The development of this bivalent vaccine has subsequently continued with the implementation of several studies. A phase II trial (55) involving 454 adults was planned to evaluate what dosage of each antigen could offer the best balance of tolerability and immunogenicity. It was found that a vaccine containing 15 μg of GI.1 VLP and 50 μg of GII.4 VLP was the best solution, as, compared to a 50 μg/50 μg preparation, it was followed by a lower incidence of adverse events (64 vs. 73%) and a higher GII.4 response, although the response to GI.1 was slightly lower. However, in both cases, immune responses to vaccination were rapid, peaking by days 7–10 and persisting through day 28.

In another study (56), various dosages of antigens and adjuvants were evaluated, and two doses of vaccine were given 28 days apart. The results confirmed that the combination of 15 μg of GI.1 VLP and 50 μg of GII.4 VLP elicited the best balance of immunogenicity with antibody titres persisting above baseline values up to 1 year after administration. However, doubts about the importance of MPL were raised, as the presence of this adjuvant, irrespective of dosage, was not associated with an increase in the immune response. In contrast, in the group of subjects given preparations containing MPL, the trend was for lower pan-Ig and HBGA-blocking antibody responses to GI.1 than that in subjects that did not receive MPL. Consequently, the formulation candidate for further studies remained the one containing 15 μg of GI.1 VLP and 50 μg of GII.4 VLP, with only aluminum as an adjuvant.

A phase II trial of this bivalent vaccine was carried out in children, toddlers and infants to evaluate the immunogenicity and safety of two doses of different amounts of the antigens (NCT02153112). A preliminary report concerning infants 6 to <12 months old and children aged 1– <4 years old revealed that all the preparations evoked a robust immune response after a single dose, with a further increase after the second dose. However, the highest the geometric mean titres (GMTs) for GI.1 and GII.4 in both age cohorts were observed after two doses of the 50 μg/150 μg formulation, with values slightly elevated in older subjects (57). Further phase II trials are ongoing or are completed even if the results are still not published. They regard immunogenicity and safety in elderly individuals (>60 years old) (NCT02661490), efficacy in adults (NCT02669121) and long-term immunogenicity in adults (NCT03039790).

#### Recombinant Adenovirus Expressing Norovirus (NoV) VP1

Guo et al. reported that the intranasal use of a vaccine based on a recombinant adenovirus expressing a GII.4 NoV VP1 could be effective in stimulating a strong systemic and mucosal specific immune response in mice, as evidenced by the detection of specific IgA and IgG in the serum, stool and intestinal and respiratory mucosa (45). Moreover, the same authors showed that when animals received vaccination with an adenovirus vector combined with a booster vaccination with NoV VLPs, the immune response could be significantly increased (58). Starting from these premises, Vaxart Inc. developed an oral NoV monovalent vaccine using a replication-defective adenovirus 5 vector expressing VP1 from a GI.1 virus. After administration to healthy adults (59), it was found to be safe and well–tolerated, as adverse events were mild or moderate. Moreover, it evoked a significant NoV GI.1-specific immune response, although the response was strictly dose related. An increase in HBGA-blocking antibody levels was evidenced in subjects receiving the higher dose (*p* = 0.0003), among whom 78% had a fold increase greater than or equal to that of the basal titer. An increase was also reported in the number of IgA+ memory B cells and fecal IgA content. However, to enhance protection offered by the recombinant adenovirus vaccine, a second similar vaccine in which the adenovirus was expressing a GII.4 VP1 was prepared and tested.

As data were completely satisfactory, Vaxart Inc. studied a combined preparation, in which an oral NoV GI.1 vaccine tablet is associated with an oral NoV GII.4 vaccine tablet that are administered concurrently. The bivalent NoV phase 1b trial includes two stages, an open-label lead-in phase that was completed successfully earlier this month, and a randomized, double-blind, placebo-controlled phase that has recently started. Both portions of the trial are designed to evaluate safety and immunogenicity, and the results are expected in a short time (60).

### Vaccines in Preclinical Development

#### Combined Vaccine for Norovirus (NoV) and Rotavirus

In an attempt to simultaneously prevent RV and NoV AGE, vaccines containing both viruses were developed. Initially, only one NoV VLP, GII.4, was included. However, as cases due to NoV GI could not be prevented, a trivalent vaccine containing two NoV VLPs (GII.4-1999 and GI.3) and the oligomeric RV VP6 was prepared by Daiichi Sankyo Company Limited & UMN Pharma Inc., Japan (61). RV VP6 was selected because it is a highly conserved protein among group A RVs, which cause 90% of all RV infections. Moreover, it is able to evoke a significant immune response that protects animals from homologous and heterologous RV infection (62). Finally, when RV VP6 is added to NoV VLPs, it can act as an adjuvant, increasing the immune response to these antigens. *In vitro* studies have demonstrated that RV VP6 facilitates NoV VLP uptake by antigen-presenting cells and improves their activation and maturation (63).

*In vivo* studies in animal models have shown that this trivalent vaccine could induce high levels of NoV- and RV-type specific serum antibodies with high avidity (>50%) and intestinal antibodies. Moreover, cross-reactive antibodies against NoV and RV types not included in the vaccine, cell-mediated immunity for both viruses and mucosal antibodies that inhibited RV were detected (64). The additional effect of VP6 was recently further evidenced in a study (65) in which the addition of RV VP6 was made to a bivalent NoV vaccine based on GI.4 and GII.4-2006a VLPs. In BALB/c mice, immunization with suboptimal doses of VLPs, alone or in combination, was not capable of evoking NoV-specific antibody production. In contrast, co-administration of VP6 led to the development of a significant immune response with high levels of homologous and heterologous antibodies.

#### Vaccines Based on P Particles

NoV vaccines based on P particles can be effective in preventing NoV infection. This fact is suggested by a comparative study (66) in which a group of neonatal animals that received an intranasal preparation of VLPs or P particles, both derived from GII.4 strain VA387, and a group of controls previously infected with the same virus were utilized. Subsequent challenge with GII.4 revealed that the risk of diarrhea was lower in vaccinated animals than in those with natural infection (82.9%), although the risk was slightly higher in animals given VLPs (46.7%) than in those receiving P particles (60%).

Moreover, the use of P particles can lead to the preparation of a number of vaccines containing antigens derived from other viruses, allowing vaccines targeting multiple potential infections. Combination vaccines for RV, hepatitis E, influenza and astrovirus have been considered. Insertion of RV VP8 into the NoV VPI P domain has been found to be able to induce potentially protective antibodies against both viruses in mice (67). Similar positive results were found when the influenza M2e gene was inserted in the NoV VPI P domain (68).

#### Combined Norovirus (NoV) and Enterovirus Vaccine

A combined vaccine effective against both NoV and enterovirus might be useful in those geographic areas where both infections are extremely common, such as the Asia-Pacific regions. A preparation containing GII.4 and EV71 VLPs was developed and compared with monovalent GII.4 and EV71 VLPs for immunogenicity in mice. The study revealed that the immune response to both antigens in subjects receiving the combination was quite similar to that obtained in individuals given the monovalent vaccines, without any immunological interference between the two antigens. EV71 infection was prevented in a similar number of tested animals, and inhibition of GII.4-VLP binding to mucin was not different (69).

## Conclusions

In recent years, identification of some NoV antigens that alone or in combination with other viral antigens can induce a potentially protective immune response has led to the development of a large series of preparations that seem capable of coping with the problems related to NoV infection. Epidemiological and immunological studies have shown that multivalent vaccines, including both GI and GII NoV, are the only solution to induce sufficiently broad protection. However, even if the road to formulation of an effective and safe NoV vaccine seems to be definitively traced, many problems still need to be solved before the total burden of NoV infections can be adequately controlled. Whether currently available vaccines are able to protect against all the heterologous NoV strains and the variants of the most common serotypes that frequently emerge and cause outbreaks must be defined. Moreover, as performed clinical trials have mainly enrolled adults, it is mandatory to know whether vaccines are effective in all age groups, including younger children. Finally, we must know the immune response of immunocompromised patients and the duration of protection induced by NoV vaccines. Only when all these problems have been solved will it be possible to establish an effective immunization schedule against NoV infection and calculate whether systematic vaccination can be cost effective. The achievement of these goals can be greatly facilitated by a more precise identification of the correlates of protection, the development of permissive cell lines for viral culture and the availability of successful animal models.

## Author Contributions

SE and NP co-wrote the manuscript and critically revised the paper and gave their scientific contribution. All authors read and approved the final version of the manuscript.

## Conflict of Interest

The authors declare that the research was conducted in the absence of any commercial or financial relationships that could be construed as a potential conflict of interest.
